# Functional analysis of *MEIS2* splice site variant *c*.*438 + 1G>T* in a congenital heart patient

**DOI:** 10.3389/fgene.2025.1629159

**Published:** 2025-09-25

**Authors:** Chenyu Xu, Junjuan Li, Xinghui Ma, Wenqi Li, Bo Jiang, Hua Bai, Fuhong Wu, Chunlian Liu, Jiwei Gu

**Affiliations:** ^1^ The General Hospital of Ningxia Medical University, Yinchuan, Ningxia, P. R. China; ^2^ Peking University First Hospital Ningxia Women and Children’s Hospital (Ningxia Hui Autonomous Region Maternal and Child Health Hospital), Yinchuan, China

**Keywords:** congenital heart disease, gene sequencing, *MEIS2*, minigene analysis, WES

## Abstract

**Aims:**

*MEIS2* (NCBI:4212; OMIM:601740) is associated with cleft palate, atrial septal defect, and varying degrees of intellectual disability. The aim of this study is to investigate the value of minigene splicing assay in the diagnosis of congenital heart disease (CHD) with mental retardation, and to explore the impact of a novel splicing-site variant on the transcript products of the *MEIS2* homeobox 2 (*MEIS2*) gene.

**Methods:**

To identify disease-causing mutations, we performed whole-exome sequencing (WES) of affected family members and subsequently employed a minigene splicing assay to evaluate the functional impact of the *MEIS2* gene splicing variant.

**Results:**

1. Postoperative transesophageal ultrasound observation of the proband showed a satisfactory umbrella shape, no shunt or displacement, and normal opening and closing of each valve; 2. WES identified a heterozygous c.438 + 1G>T variant in the intronic region of the *MEIS2* gene, which was a *de novo* mutation confirmed by Sanger sequencing; 3. The results of the minigene splicing assay showed that c.438 + 1G>T affected the normal splicing of precursor mRNA. This was demonstrated consistently by constructing pcDNA3. 1 and pcMINI-C vectors. Two aberrant splicing modes were identified after the mutation: ① Retention of 290 bp in intron4, resulting in a frameshift and the introduction of a premature termination codon (PTC) within the retained fragment, predicted to produce a truncated protein of 175 aa (p.Met147Leufs*30); ② Exon4 skipping, represented at the cDNA and protein levels as c.388_438del p. Val130_Leu146del, which did not cause a frameshift but led to an internal deletion of 17 aa within the protein, predicted to result in a truncated protein of 460 aa.

**Conclusions:**

Minigene splicing assay revealed a new molecular marker for the definitive diagnosis and genetic counseling of CHD. Functional analysis to verify intronic pathogenicity has important diagnostic value. The study expanded the *MEIS2* genetic spectrum and provided laboratory evidence for clinical diagnosis and treatment.

## 1 Introduction

CHD is a relatively common congenital condition in newborns and the leading cause of death related to birth defects, with a prevalence of approximately 0.8%–1.2% (Tennant et al., 2010; Wu et al., 2020). Its etiology is complex, involving genetic, non-genetic, and environmental factors, among which emerging genetic variants play a particularly important role (Lee et al., 2021; Magnan et al., 2024; Grunert et al., 2016; Hu et al., 2018; Øyen et al., 2009; Jarrell et al., 2019). In this study, a child with CHD was admitted for surgical treatment. Whole-exome sequencing (WES) and Sanger sequencing revealed a heterozygous mutation in the *MEIS2* gene, *c*.*438 + 1G>T* (nucleotide 438 is the last nucleotide (G) of exon4, transversion occurs at the splice donor site of intron4, where G is replaced by T), which is a splicing mutation located in intron4. This mutation has not been recorded in the National Center for Biotechnology Information Clinical Mutation Database (ClinVar) or the Human Gene Mutation Database (HGMD). According to the 2015 classification guidelines for genetic variation from the American College of Medical Genetics and Genomics (ACMG), the mutation was classified as pathogenic, although functional data were not available. In this study, functional analysis of *MEIS2 c*.*438 + 1G>T* was conducted to verify its pathogenicity and association with cardiac malformation.

## 2 Materials and methods

### 2.1 Clinical information

**FIGURE 1:**
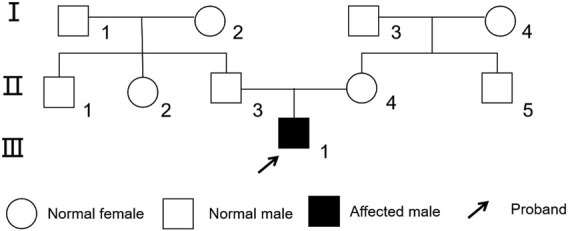
Family tree of the proband with congenital heart disease. The proband was indicated by an arrow.

We selected a patient with CHD and developmental delay who was admitted to the Department of Cardiovascular Surgery, General Hospital of Ningxia Medical University in February 2023. The male patient, aged 1 year and 8 months, was born in China and was of Han ethnicity, had undergone unilateral inguinal cryptorchidism surgery at 10 months. Echocardiography revealed CHD and a secundum atrial septal defect. The child exhibited developmental and language delays, slow response, low activity, and poor coordination (The Bailey III score was 78 points, indicating at least mild to moderate impairment). Physical examination revealed systolic murmurs in the second and third intercostal spaces along the left sternal border. The family reported no history of genetic disease. The mother was 25 years old (gravida 1, para 1), and the father was also 25 years old. The parents were not consanguineous. The family pedigree was shown in Figure 1. After obtaining informed consent, 5 mL of peripheral venous blood was collected from the proband and both parents for WES and Sanger sequencing. Subsequently, we conducted WES testing on 30 patients with heart-related diseases and 100 healthy individuals, and no *MEIS2* gene *c*.*438 + 1G>T* gene mutations were found. The study was approved by the Ethics Committee of the Reproductive Medicine Center, General Hospital of Ningxia Medical University (Approval No:SZLL20230202).

### 2.2 Genetic analysis

Genomic *DNA* was extracted using the. *DNA* was extracted using a QIAamp *DNA* Blood Midi/Mini Kit (Cat No.51185 Qiagen GmbH, Hilden, Germany) following the manufacturer’s instructions. *DNA* concentration and purity were measured using the Qubit 4.0 Fluorometer (Thermo Fisher Scientific, United States). A total of 1.5 μg of genomic *DNA* was used to construct the sequencing library. *DNA* fragmentation, end repair, and adaptor ligation were performed using the HieffNGS OnePot *DNA* Library Prep Kit for Illumina. Whole-exome library construction was carried out using the NanoWES system (Berry Genomics, Beijing, China). Paired-end sequencing was performed on the NovaSeq 6,000 platform (Illumina, San Diego, United States) using PE150 mode. Sequencing data were filtered and evaluated, with low-quality reads (Q30 < 85%) excluded. Reads were aligned to the human reference genome (UCSC hg19, https://genome.ucsc.edu/) using Burrows-Wheeler Aligner (BWA; https://bio-bwa.sourceforge.net/). *PCR* repetitive sequence markers, Re-calibration of base quality values, Variant detection, Variant quality control and filtering were analyzed by the Genome Analysis Toolkit (GATK; https://software.broadinstitute.org/gatk/) and the Verita TrekkerVariants Detection System (Berry Genomics). Variants were annotated and functionally predicted using databases such as dbSNP, 1,000 Genomes, ClinVar, and tools including SIFT, PolyPhen-2, and GERP. Mutation sites in family members were validated using Sanger sequencing. *MEIS2* primers were designed using Primer Z software. *PCR* amplification was performed under specific primer conditions, and the products were analyzed using 1% agarose gel electrophoresis for identification, purification, and recovery. The purified *PCR* products were subjected to Sanger sequencing (ABI 3730). Data were analyzed after sequencing was completed. In addition, variable splicing predictions were conducted using software such as Rare Disease Data Center (RDDC, https://rddc.tsinghua-gd.org/), SpliceAI (https://spliceailookup.broadinstitute.org).

### 2.3 Minigene analysis

The wild-type (*WT*) and mutant-type (*MT*) forms of the minigene regions, encompassing Exon3 (142bp)-Intron3 (674bp)-Exon4 (51bp)-Intron4 (986bp)-Exon5 (51bp)of *MEIS2*, were amplified from genomic *DNA* of the proband, using the following primer pairs: *5′- TTG​GCC​TCG​TCT​GAA​ATG​CCC-3′* and *5′-TCT​GGC​CAG​ATC​TGA​GGG​AC-3′, 5′-TGCCTGAGGGG AATATTGGG-3′* and *5′-TGG​AGC​TGG​GGA​GAG​CAT​TA-3′*. The amplified products were respectively cloned into the *pcDNA3*.*1* and *pcMINI-C* vectors (Figure 5A). The results of the selected clones were verified using colony/bacterial liquid PCR and Sanger sequencing. The recombinant vectors were transiently transfected into *HeLa* and *293T* cells. Cells were collected 48 h after transfection. For minigene transcription analysis, total *RNA* was extracted from transfected cells, and *RNA* concentrations were measured. *cDNA* was synthesized by reverse transcription using equal amounts of *RNA*. *pcDNA3*.*1-WT/MT* was amplified using primers *pcDNA3*.*1-F (CTAGAGAACCCACTGCTTAC)/pcDNA3*.*1-R (TAG​AAG​GCA​CAG​TCG​AGG*), and *pcMINI-C-WT/MT* was amplified using primers *pcMINI-C-F (ACTTAAGCTTatgagtgggctttggggtggccggtt*)*/pcMINI-C-R* (*TAG​AAG​GCA​CAG​TCG​AGG*). *PCR* products were analyzed by agarose gel electrophoresis. Each band was recovered and subjected to Sanger sequencing. Sequencing confirmed successful insertion of both *WT* and *MT* minigenes into the corresponding vectors. The nucleotide sequences were translated into protein sequences and analyzed to assess the effect of the mutation on transcription and translation.

## 3 Results

### 3.1 Surgical treatment

**FIGURE 2:**
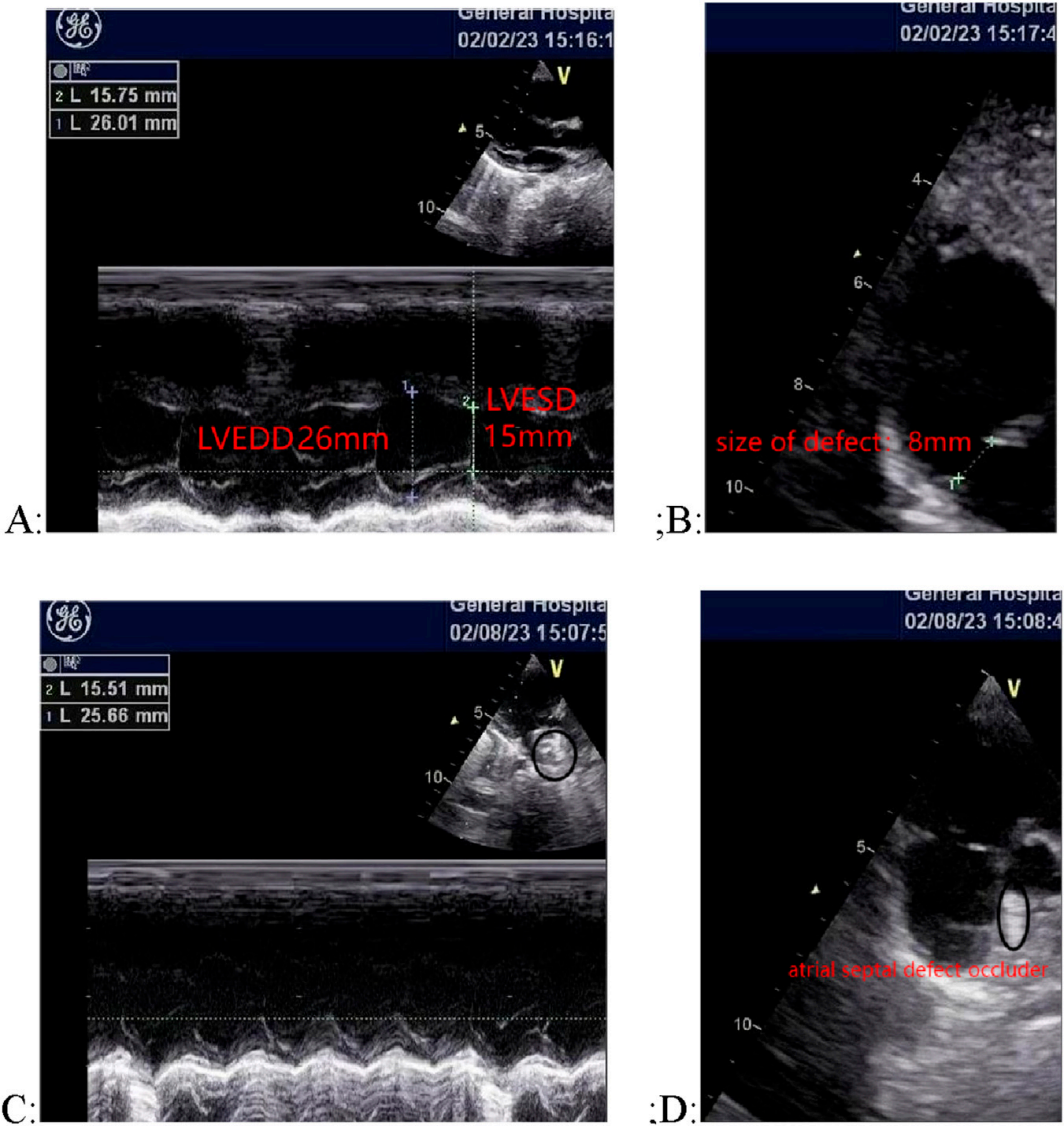
Comparison of cardiac ultrasound images before and after surgery. **(A)** and **(B)** Preoperative ultrasound images, showing an 8 mm atrial septal defect; **(C)** and **(D)** Postoperative ultrasound images, showing the occluder in place with no shunt or displacement.

The patient underwent closure surgery after evaluation. Transesophageal ultrasound showed that the atrial septal defect measured 8 mm during the procedure. A 3 cm incision was made in the right third intercostal space to access the chest. The pericardium was exposed and incised. A double-layer purse-string suture was placed in the right atrium using 5–0 polypropylene suture. Under ultrasound guidance, the needle sheath was punctured, and a 14 mm atrial septal defect occluder was successfully implanted. The push-pull test showed stable positioning. The occluder had a satisfactory umbrella shape with no shunt or displacement, and it was adjusted and released under transesophageal ultrasound monitoring. The heart valves opened and closed normally. The patient’s preoperative and postoperative oesophageal ultrasound is shown in Figure 2.

### 3.2 Sanger sequencing analysis of *MEIS2 c.438 + 1G>T*

**FIGURE 3:**
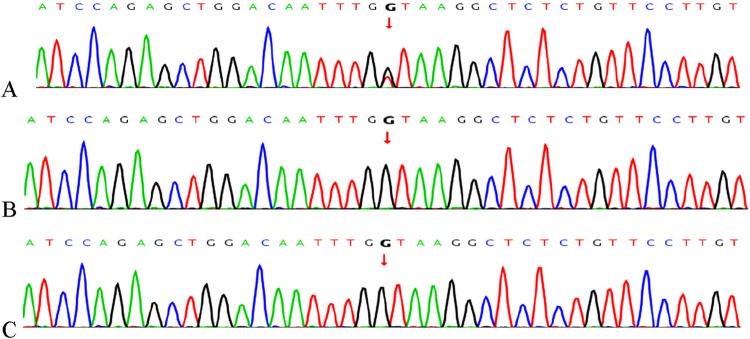
Gene mutations in the child and parents confirmed by Sanger sequencing. **(A)** The *c*.*438 + 1G>T* variant in *MEIS2* identified in the proband; **(B)** Father; **(C)** Mother.

A heterozygous mutation, *c*.*438 + 1G>T*, in the *MEIS2* gene was detected by WES. This splicing mutation was located in intron 4 of the *MEIS2* gene (See Figure 3 for details). The variant has not been recorded in the ClinVar or HGMD databases to date. According to the ACMG classification guidelines for genetic variation (2015 edition), the mutation was classified as pathogenic (PVS1 + PM2 + PM6_Supporting). This site had not been previously reported and is described here for the first time.

### 3.3 The pathogenic *MEIS2 c.438 + 1G>T* gene variant

**FIGURE 4:**
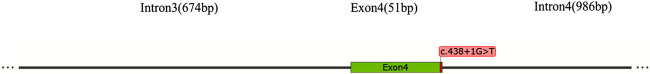
Schematic diagram of the mutational position in *MEIS2* introns.

The *MEIS2* gene contains 12 exons and encodes a protein of 477 amino acids. The *c*.*438 + 1G>T* mutation is located at the + 1 position of intron4, where the base at position *c*.*438 + 1* is replaced G by T (As shown in Figure 4). This site lies within the “Class I mutation region” that affects splicing. The mutation was predicted using three bioinformatics databases—RDDC (Prediction Score:0.9937), SpliceAI (Score:1.00), and FF—all of which indicated that the mutation disrupts splicing.

**FIGURE 5:**
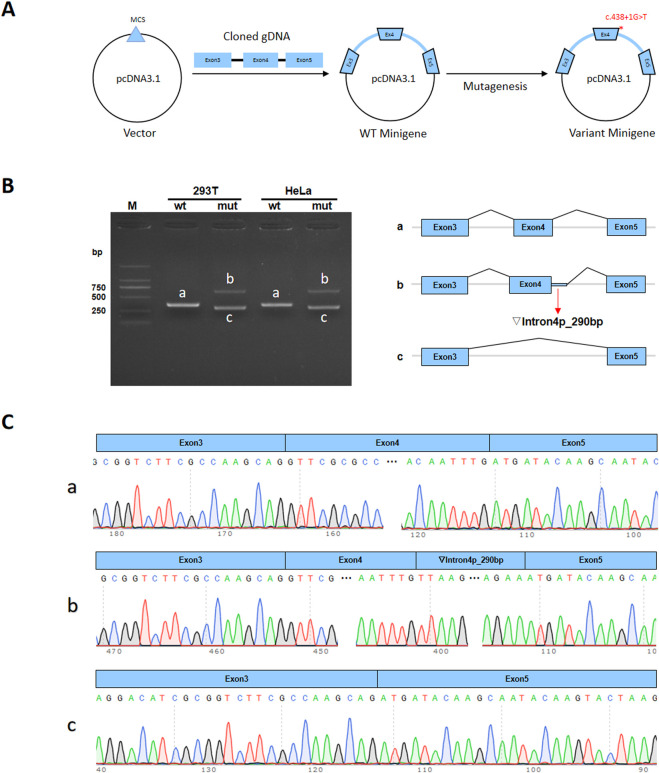
Minigene assay of the *MEIS2 c*.*438 + 1G>T* variant and schematic diagram of splicing patterns. **(A)** Minigene vector construction; **(B)** *RT-PCR* gel electrophoresis showing one band for wild-type and two bands for mutant-type. Bands in *HeLa* and *293T* cells are labeled **(a–c)** **(C)** Sanger sequencing confirmed that wild-type minigene produced normal *mRNA*.

*In vitro* results from the minigene assay demonstrated that the *c*.*438 + 1G>T* mutation affects normal *mRNA* splicing. This was consistently shown using *pcDNA3. 1* and *pcMINI-C* vectors. *RT-PCR* results revealed a single band for wild-type (*WT*) and two bands for mutant-type (*MT*) in both *HeLa* and *293T* cells (Figure 5B). Sanger sequencing showed normal splicing for *WT*, while *MT* showed abnormal splicing, including intron retention and exon skipping (Figure 5C). The mutation led to two abnormal splicing patterns: ① Retention of 290 bp at the 5′end of intron4, expressed at the *cDNA* and protein level as *c*.*438 + 1_438 + 290ins*; ② Exon4 skipping, expressed as *c*.*388_438del*.

### 3.4 Bioinformatic analysis

**FIGURE 6:**
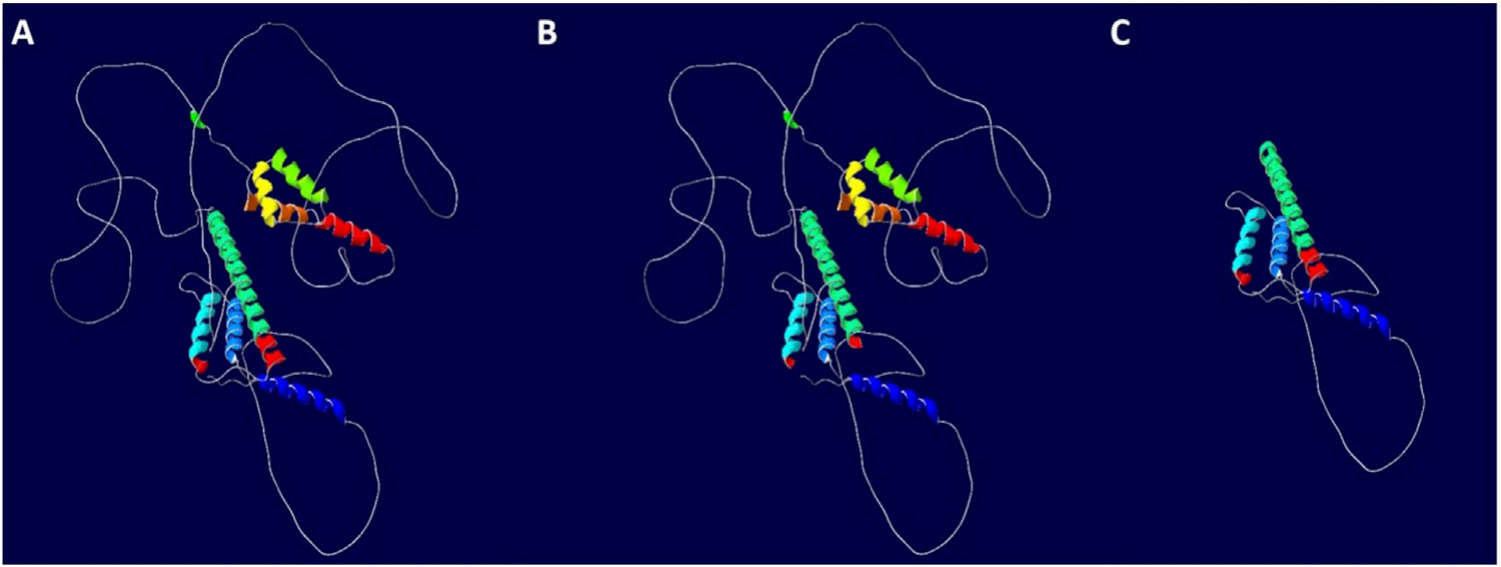
Impact of the *MEIS2 c*.*438 + 1G>T* variant on downstream protein translation. **(A)** WT protein; **(B)** Mutant 1 (Exon4 skipping); **(C)** Mutant 2 (Intron4 retention).

To further evaluate the impact of intron retention and exon skipping on *MEIS2* protein translation, bioinformatics analysis was performed. The results showed the following: ① The 290 bp retention at the 5′end of intron4 caused a frameshift, introducing a premature termination codon (PTC) within the retained fragment. The location of PTC on the chromosome is Chr15:37094574–37095476. Predicted to result in a truncated protein of 175 amino acids (*p*.Met147Leufs*30). ② Exon4 skipping did not cause a frameshift but led to an internal deletion of 17 amino acids. This predicted to produce a truncated protein of 460 amino acids, suggesting that the mutation disrupted the normal gene splicing process (See Figure 6 for details).

## 4 Discussion

Studies have confirmed that mutant genes causing CHD primarily play key regulatory roles during the early stages of heart development. These genes include cardiac transcription factors, heart-specific genes, and signaling pathway molecules (Warrington et al., 2019). Several critical genes such as *GATA4, IRX4, TBX5, *MEIS2*, ILS1*, and *NKX2-5* are involved in the differentiation of progenitor cells into cardiovascular lineages and play essential roles in cardiac morphogenesis (Magnan et al., 2024; Morton et al., 2022).

The human *MEIS2* gene is located on chromosome 15q14. *MEIS2* belongs to the TALE family of transcription factors and encodes a protein containing a homeodoma-in (Verheije et al., 2019). It is associated with an autosomal dominant genetic disorder. Researchers constructed a *MEIS2* conditional mutant mouse model and found that systemic inactivation of the *MEIS2* gene leads to lethality on embryonic day 14. They discovered that neural crest cells express *MEIS2*, and embryos with *MEIS2* deficiency exhibit defects in tissues derived from neural crest cells. *MEIS2*-deficient mice are embryonic lethal. The experiment reveals the critical role of *MEIS2* in the development of cranial facial and cardiac neural crest cells in mice (Machon et al., 2015). Previous functional studies have shown that *MEIS2B*, one of the two homologous genes of *MEIS2* in zebrafish, plays an important role in cardiac formation, regulation, and function. Knockdown of *MEIS2B* in zebrafish embryos leads to defects in cardiac morphogenesis, including the absence of midline formation of the linear heart tube, severe cardiac looping defects, pericardial effusion, and a significantly reduced heart rate. The expression pattern of *MEIS2B* in the cardiac region of developing mutant zebrafish embryos is highly similar to that of the known cardiac transcription factor GATA4. This case report describes a patient with cleft palate and cardiac septal defect, in whom a three-base-pair non-frameshift deletion (*c.998_1000del:p.Arg333del*) in the homeodomain of the *MEIS2* gene was identified, the deletion of the arginine residue may have an additional dominant-negative effect (Hu et al., 2020). Regarding intellectual disability and developmental delay, *MEIS2B* is expressed in the hindbrain segments during development, and *MEIS2* is a key factor in hindbrain patterning. Researchers indicated that *CDK2*, *RAD51*, *BRCA1*, and *MCM3* were expected to become new therapeutic targets for neuroblastoma patients with *MEIS2* deficiency (Louw et al., 2015).

Pathogenic variants in *MEIS2* typically cause three core clinical features: cleft palate, atrial and ventricular septal defects, and developmental delay. Additional phenotypes have been reported, including primary neutropenia, branchial anomalies, and complex genital anomalies (Su et al., 2021). In recent years, an increasing number of novel phenotypes have been successively discovered and documented, Researchers collected 23 previously unreported patients, among whom 9 had *de novo* sequence variations in the *MEIS2* gene, and 14 had 15q14 microdeletions affecting *MEIS2*. In addition to the classic triad of malformations heterozygous deletion of *MEIS2* also leads to g thin and arched eyebrows, short alae nasi, and thin vermillion. A comparison of genotype-phenotype between patients with 15q14 deletions and those with intragenic sequence variations or intronic deletions in *MEIS2* revealed a higher prevalence of moderate to severe intellectual disability in the former group, suggesting the presence of an independent psychomotor development-related gene locus near the *MEIS2* gene (Verheije et al., 2019). Fujita described clinical manifestations included severe intellectual disability, moderate delays in motor and language development, cleft palate, ventricular septal defect, hyperopia, severe feeding difficulties accompanied by gastroesophageal reflux, and constipation. Notably, feeding difficulties with concurrent gastroesophageal reflux have been recognized as one of the core clinical features in cases of heterozygous *MEIS2* gene deletion (Fujita et al., 2016).

In recent years, some studies have expanded the known mutation spectrum of *MEIS2*. More than 20 individuals with *de novo* pathogenic variants in *MEIS2* have been described in the literature. Andrea G reported a *de novo* missense variant, *MEIS2c*.*998G>A*;*p*.*Arg333Lys* (Gangfuß et al., 2021). Douglas (Douglas et al., 2018)identified missense variants *Pro302*-L*eu*, *Gln322Leu*, *Arg331Lys*, and *Val335Ala* located within the functional homeodomain of *MEIS2* by WES. Fujita described a female patient harboring a *de novo* nonsense mutation in the *MEIS2* gene (c.611C>G, p. Ser204*) (Verheije et al., 2019). In this study, functional analyses were conducted to assess the pathogenicity of an splice site variant in the *MEIS2* gene. A novel pathogenic intronic variant, *c*.*438 + 1G>T*, located at the + 1 position of intron4, was added to the *MEIS2* mutational spectrum. According to the ACMG classification guidelines for genetic variation (2015 edition), the mutation was classified as pathogenic (PVS1 + PS3 + PM2 + PM6_Supporting, post-assay). Shear prediction using the RDDC bioinformatics database showed that this mutation affected splicing function, with a positive prediction rate of 90%. The minigene splicing assay demonstrated that the mutation led to two abnormal splicing patterns: retention of 290 bp at the 5′end of intron4 and skipping of exon4. These abnormalities interfered with downstream translation and resulted in protein truncation. Intron retention in the mature transcript directly reflects the influence of multiple intrinsic and extrinsic regulatory factors (Monteuuis et al., 2019). Studies have shown that minor intron retention contributes to disease susceptibility (Inoue et al., 2021; Monteuuis et al., 2021). Exon skipping is the most common alternative splicing event, often leading to loss of functional domains or frameshift mutations. It contributes to a wide range of human diseases and has been considered a therapeutic target (Han et al., 2023; Mazieres et al., 2023). The selection of HeLa and 293T cell lines in this study was primarily based on their widespread application in splicing mechanism research, high transfection efficiency, and convenience as preliminary screening tools. The primary objective was to efficiently detect whether mutations cause fundamental splicing defects (such as exon skipping, intron retention, etc.). However, the observed splicing patterns may not fully represent the precise regulation of *MEIS2* in cardiac tissue, particularly in developing hearts. To more accurately simulate the impact of this gene on cardiac function, the use of cardiac-specific cell lines would be more appropriate, such as *H9c2* cardiomyocytes or induced pluripotent stem cell (*iPSC*) derived cardiomyocytes. This would provide a more targeted platform for studying the cardiac-related effects of *MEIS2* gene mutations.

As a cardiac surgeon, beyond performing surgery, providing recurrence risk counseling to families is of great importance. In this case, the pathogenic cause was identified through WES. The proband’s parents were advised to initiate physical rehabilitation early to improve the child’s functional status. Genetic counseling should include careful screening of the maternal family history of CHD to rule out mosaicism in unaffected parents (Zhang et al., 2021; Slaba et al., 2023). Prenatal diagnosis is recommended to reduce the risk of heritable mutations and new birth defects in future offspring. This study expanded the *MEIS2* gene mutation spectrum through analysis of a CHD patient and emphasized that cardiac surgeons not only repair anatomical defects but also contribute to identifying pathogenic mutations. This approach supports early diagnosis, guides rehabilitation, and informs reproductive decisions, demonstrating significant clinical value.

## Data Availability

The data presented in the study are deposited in the National center for biotechnology information repository, accession number is PRJNA1327239.
